# Low temperature ionic conductor: ionic liquid incorporated within a metal–organic framework†
†Electronic supplementary information (ESI) available: Experimental details, structures of ZIF-8 and EMI-TFSA, TEM image of ZIF-8 nanocrystals, results of structural characterizations, Nyquist plots, IR spectra and results of elemental analysis. See DOI: 10.1039/c5sc01398d



**DOI:** 10.1039/c5sc01398d

**Published:** 2015-05-05

**Authors:** Kazuyuki Fujie, Kazuya Otsubo, Ryuichi Ikeda, Teppei Yamada, Hiroshi Kitagawa

**Affiliations:** a R&D Center Kagoshima , Kyocera Corporation , 1-4 Kokubuyamashita-cho , Kirishima-shi , Kagoshima 899-4312 , Japan . Email: kazuyuki.fujie.hs@kyocera.jp; b Division of Chemistry , Graduate School of Science , Kyoto University , Kitashirakawa Oiwake-cho , Sakyo-ku , Kyoto 606-8502 , Japan . Email: kitagawa@kuchem.kyoto-u.ac.jp; c Department of Chemistry , University of Tsukuba , Tsukuba 305 , Japan; d Core Research for Evolutional Science and Technology (CREST) , Japan Science and Technology Agency (JST) , 5 Sanban-cho , Chiyoda-ku , Tokyo 102-0075 , Japan; e Institute for Integrated Cell-Material Sciences (iCeMS) , Kyoto University , Yoshida , Sakyo-ku , Kyoto 606-8501 , Japan; f INAMORI Frontier Research Center , Kyushu University , 744 Motooka , Nishi-ku , Fukuoka 819-3095 , Japan

## Abstract

Ionic liquids (ILs) show promise as safe electrolytes for electrochemical devices. However, the conductivity of ILs decreases markedly at low temperatures because of strong interactions arising between the component ions. Metal–organic frameworks (MOFs) are appropriate microporous host materials that can control the dynamics of ILs *via* the nanosizing of ILs and tunable interactions of MOFs with the guest ILs. Here, for the first time, we report on the ionic conductivity of an IL incorporated within a MOF. The system studied consisted of EMI-TFSA (1-ethyl-3-methylimidazolium bis(trifluoromethylsulfonyl)amide) and ZIF-8 (Zn(MeIM)_2_, H(MeIM) = 2-methylimidazole) as the IL and the MOF, respectively. While the ionic conductivity of bulk EMI-TFSA showed a sharp decrease arising from freezing, the EMI-TFSA@ZIF-8 showed no marked decrease because there was no phase transition. The ionic conductivity of EMI-TFSA@ZIF-8 was higher than that of bulk EMI-TFSA below 250 K. This result points towards a novel method by which to design electrolytes for electrochemical devices such as batteries that can operate at low temperatures.

## Abstract 2

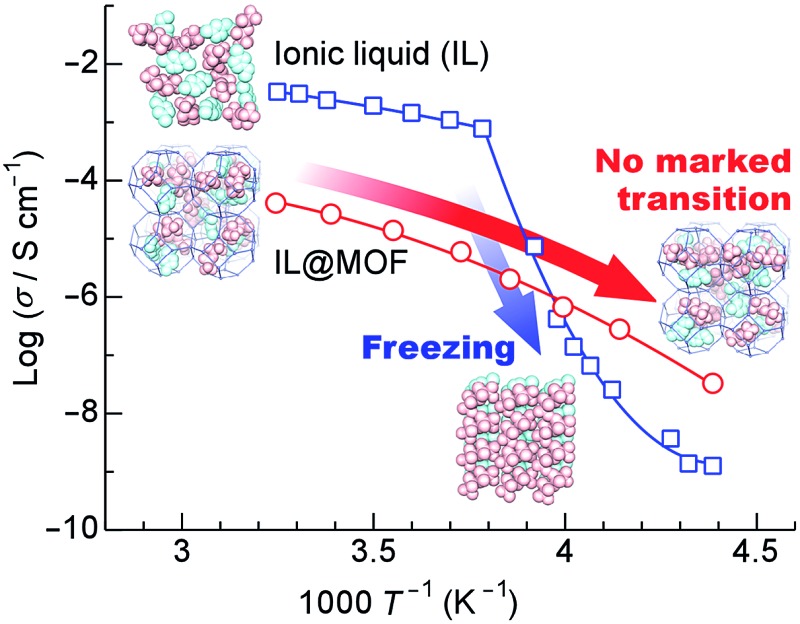
An ionic liquid incorporated into micropores of a metal–organic framework showed higher ionic conductivity than bulk ionic liquid at low temperature because of the absence of marked freezing transition.

## Introduction

Ionic conducting materials have increasingly gained importance in recent years for applications in electrical energy storage and generation. They are used as electrolytes in electrochemical devices, such as secondary batteries, electric double layer capacitors (EDLCs), and fuel cells. These electrochemical devices should operate at low temperatures of 253 K or below for automotive applications, such as electric or hybrid electric vehicles, since vehicles may be exposed to these temperatures.^1^ Therefore, these devices, particularly lithium ion batteries and EDLCs, contain volatile and flammable organic solvents as electrolytes to avoid freezing of the electrolyte and a decrease in ionic conductivity at low temperatures.^2^

In order to avoid the use of these flammable solvents, ionic liquids (ILs) are promising candidate materials for safe electrolytes in electrochemical devices.^3^ ILs have desirable properties, such as non-flammability, negligible volatility, high electrochemical and thermal stability, and high ionic conductivity. However, the ionic conductivity of ILs is very low at low temperatures,^4^ at which the mobility of the ions decreases markedly owing to the formation of intermolecular interactions,^5^ which are particularly strong below the freezing point of the IL. Being able to tune the intermolecular interactions of the ions is a significant issue for controlling the ionic conductivity and phase behavior of ILs.

Metal–organic frameworks (MOFs) are supposed to be desirable host materials for this purpose. MOFs are a novel group of highly porous, crystalline materials with regular, well-defined micropores. The properties of various types of MOFs have been studied, such as gas adsorption^6^ and separation;^7^ their catalytic,^8^ magnetic,^9^ electronic,^10^ and optical^11^ properties; and their ionic conductivity.^12^ Furthermore, MOFs can be designed using several different characteristics, such as pore size, framework topology, surface area, and interactions with guest molecules. Therefore, MOFs are appropriate materials for controlling the dynamics of small molecules^13^ such as ILs *via* the nanosizing of ILs and tunable interactions of MOFs with the guest ILs.

We have reported previously that an IL inside a MOF's micropores (denoted as IL@MOF) shows no marked phase transition owing to the nanosizing of the IL, even in the low temperature region,^14^ even though the bulk IL showed the usual freezing and melting behavior. This observation suggests that IL@MOF is a promising ionic conductor that could work at low temperatures. Here, we demonstrate that ionic conduction occurs in the IL@MOF system. We used a thermally and chemically stable MOF, ZIF-8 (Zn(MeIM)_2_, H(MeIM) = 2-methylimidazole),^15^ and a highly conductive IL, EMI-TFSA (1-ethyl-3-methylimidazolium bis(trifluoromethylsulfonyl)amide).

## Experimental

We incorporated EMI-TFSA into the ZIF-8 nanoparticles (denoted as EMI-TFSA@ZIF-8) by mixing the two materials together using a mortar and pestle, where the EMI-TFSA could theoretically occupy the micropore volumes in the ZIF-8 at loadings of 25%, 50%, 75%, 100%, and 125% (denoted as EZ25, EZ50, EZ75, EZ100, and EZ125, respectively). The smaller particle size of ZIF-8 is preferred to introduce EMI-TFSA into the central portion of each ZIF-8 particle. The mixtures were heated and stored overnight to enhance the diffusion of EMI-TFSA into the micropores. Sample preparation and analysis, except for X-ray powder diffraction (XRPD) experiments, were carried out under inert conditions to prevent water adsorption and absorption.

## Results and discussion

**Fig. 1:**
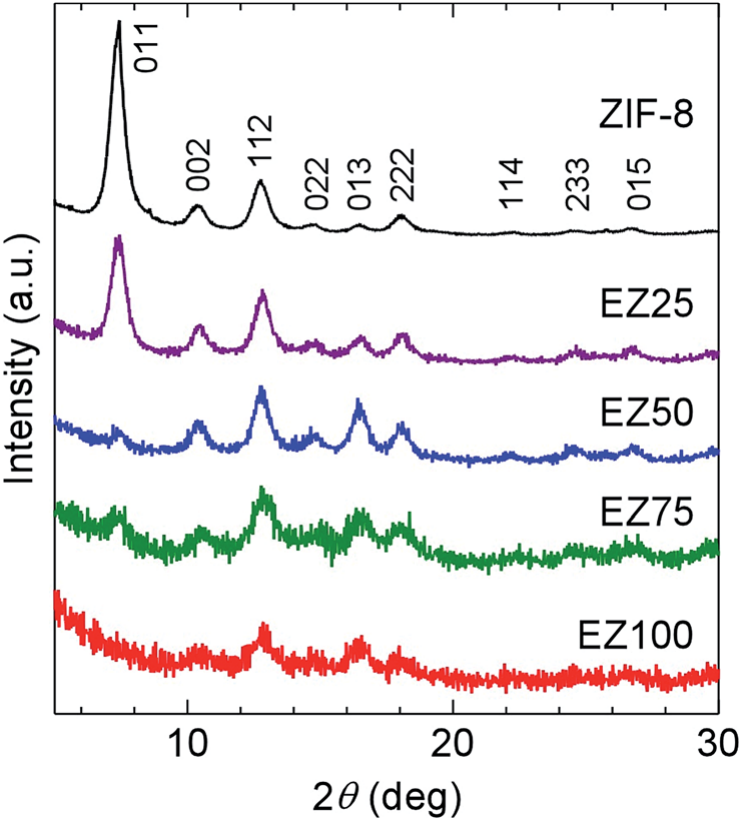
XRPD patterns of ZIF-8, EZ25, EZ50, EZ75, and EZ100. The plane indices were assigned with reference to a previous report.^15^

As shown in the XRPD patterns in Fig. 1, the synthesized ZIF-8 were crystalline, and showed very broad and weak diffraction because of small crystallite size. The crystal size of the ZIF-8 nanocrystals was estimated to be 12 nm using Scherrer's equation. The ZIF-8 crystal structure remained stable, even after mixing with EMI-TFSA and subsequent heating. The relative intensities of the peaks changed on incorporation of the EMI-TFSA. A similar change in relative intensity was reported in alkylammonium salt included ZIF-8.^16^

**Fig. 2:**
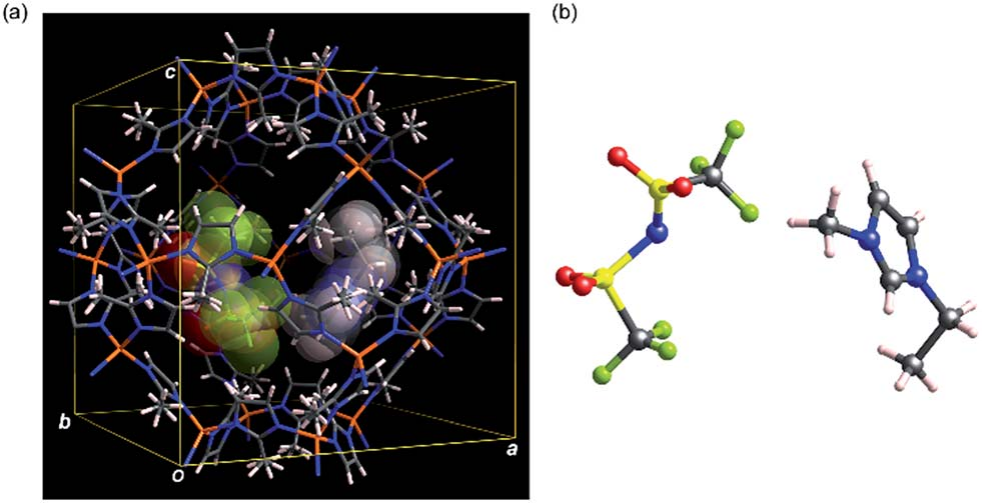
Model crystal structure (a) and an EMI-TFSA ion pair inside the pore (b) of EZ25 based on the Rietveld refinement in the bulk state. In both panels, one pair of EMI^+^ and TFSA^–^ extracted from disordered components is shown for clarity. In panel (a), included EMI-TFSA units are drawn as the stick model with superimposed CPK model. Zinc, carbon, nitrogen, oxygen, fluorine, sulfur and hydrogen atoms are shown in orange, grey, blue, red, green, yellow, and pink, respectively.

To obtain structural information of EMI-TFSA loaded ZIF-8 samples, structural analyses were carried out using the simulated annealing (SA) method installed on EXPO2013 (ref. 17) followed by Rietveld refinement using the RIETAN-FP^18^ program. We also investigated electron density within the micropores of the present materials using the maximum entropy method (MEM) using the Dysnomia^19^ program (for details, see ESI†). Only for these structural analyses, bulk samples of ZIF-8 (ZIF-8_bulk_: commercially available Basolite® Z1200) and EMI-TFSA loaded ZIF-8_bulk_ with varying loading amount (EZ25_bulk_ and EZ100_bulk_)^14^ were used (Fig. S4†). The calculated diffraction patterns based on the Rietveld refinements are in good agreement with experimental patterns indicating that EMI-TFSA units are surely included inside the cage of ZIF-8 (Fig. S5 and S6†). The model crystal structure obtained from Rietveld refinement of EZ25_bulk_ is shown in Fig. 2 (see also Fig. S7†). From the MEM analysis, no obvious charge density was found within the micropore of ZIF-8_bulk_ indicating that the ZIF-8_bulk_ contains no guest molecules. In contrast, apparent electron density peaks were found within the micropores of EZ25_bulk_ and EZ100_bulk_ (Fig. S5 and S6†). In addition, the results of MEM analyses also show that the charge density originating from EMI-TFSA units was low at the centre of the micropore suggesting that EMI-TFSA units attractively interact with the host ZIF-8 framework (Fig. S8†).

We carried out elemental analysis for ZIF-8, EZ50, and EZ100 (Table S1†). The observed CHN ratios are almost the same as the calculated values based on the molar ratio in preparation. This result indicates that the molar ratio of EMI-TFSA to ZIF-8 remains unchanged even after mixing and subsequent heating, because of negligible volatility of the EMI-TFSA.

**Fig. 3:**
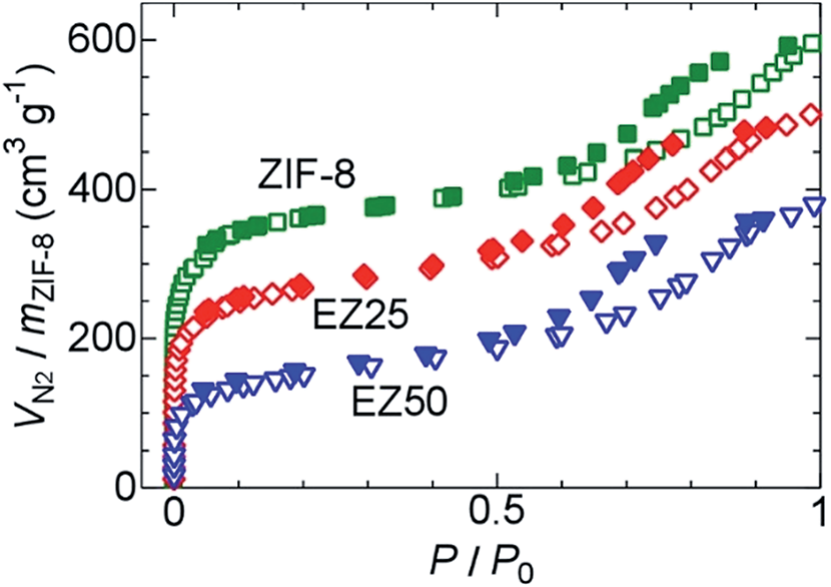
Nitrogen gas adsorption and desorption isotherms of ZIF-8 (green), EZ25 (red), and EZ50 (blue) at 77 K. The open and closed symbols indicate adsorption and desorption, respectively.

Nitrogen gas adsorption measurements were carried out to confirm the existence of EMI-TFSA inside the ZIF-8 micropores. As shown in Fig. 3, the samples of ZIF-8, EZ25, and EZ50 showed a sharp uptake at low relative pressure, and a gradual uptake exhibiting hysteresis at higher relative pressure, with the former and the latter values indicating the existence of micropores and mesopores, respectively. The micropores originate from the ZIF-8 framework. The mesopores originate from adsorption in the intergranular spaces between agglomerated ZIF-8 nanoparticles. Agglomeration and fusion of the ZIF-8 nanoparticles was observed using transmission electron microscopy (TEM), as shown in Fig. S3.† The nitrogen gas uptake at low relative pressure decreased with increasing concentration of introduced EMI-TFSA, indicating that the EMI-TFSA was preferentially introduced into the micropores rather than the mesopores.

**Fig. 4:**
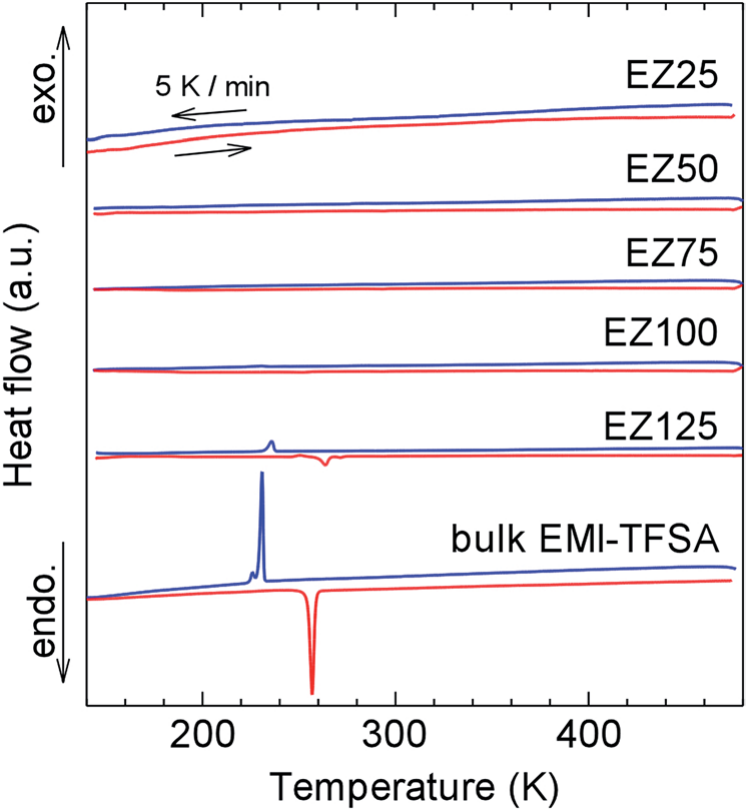
DSC curves of EZ25, EZ50, EZ75, EZ100, EZ125, and bulk EMI-TFSA. The red and blue lines indicate heating and cooling, respectively, at a fixed scan rate of 5 K min^–1^.

Differential scanning calorimetry (DSC) measurements were conducted (Fig. 4) to study phase transitions in the nanosized EMI-TFSA. Bulk EMI-TFSA showed sharp peaks occurring at 257 K on heating and at 231 K on cooling, which indicated melting and freezing of the sample, respectively. By contrast, EMI-TFSA@ZIF-8 except for EZ125 showed no peaks in the DSC measurements between 143 and 473 K. Weak anomalies appeared at almost the same temperatures of the melting and freezing of the bulk EMI-TFSA only in EZ125. This result can be explained by the melting and freezing of the excess EMI-TFSA that was located outside the micropores of ZIF-8. The absence of peaks in EZ25, EZ50, EZ75, and EZ100 suggests that the nanosized EMI-TFSA in the micropores of ZIF-8 was prevented from freezing. Using the pore diameter of ZIF-8 (ref. 15) and the van der Waals volumes of the EMI^+^ cations and TFSA^–^ anions,^20^ we confirmed that the storage capacity of each ZIF-8 micropore was only three ion pairs or fewer. This small number of ions is not enough to construct an ordered crystal structure.

**Fig. 5:**
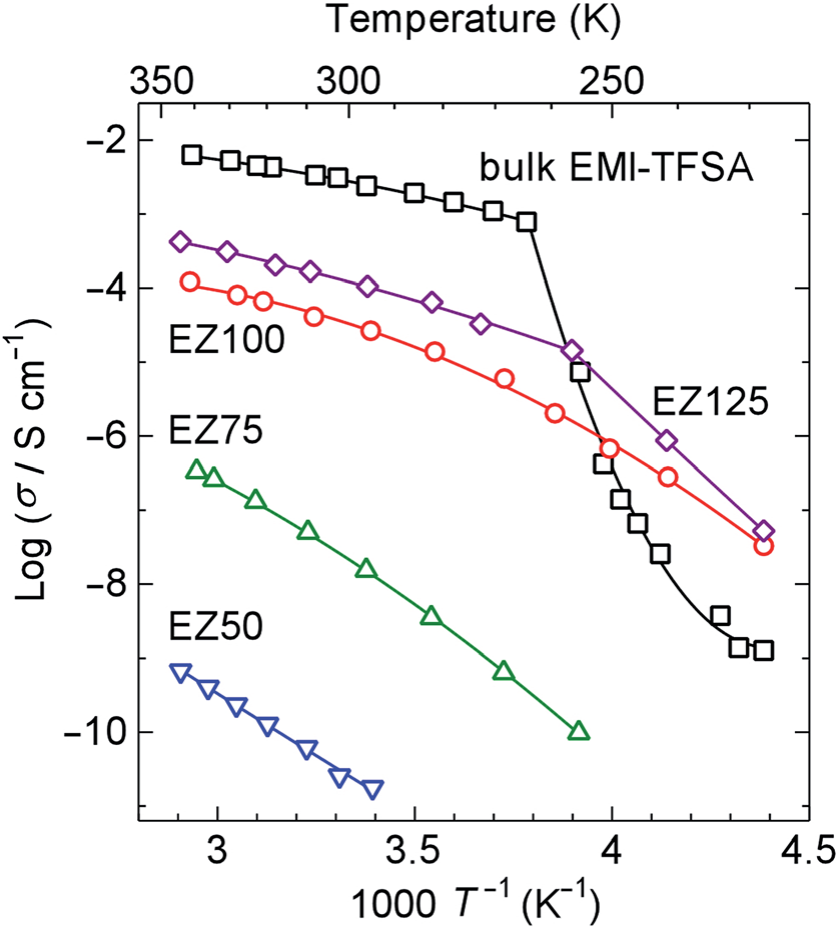
Arrhenius plots of the ionic conductivity of EZ50, EZ75, EZ100, EZ125, and bulk EMI-TFSA on heating. The solid lines are provided as guides for the eye.

Fig. 5 shows Arrhenius plots of the ionic conductivity. Bulk EMI-TFSA exhibited a sharp decrease in conductivity below 264 K. EZ125, which has the excess EMI-TFSA outside of the micropores of ZIF-8, also showed a decrease in conductivity with the inflection point around 257 K. These temperatures correspond to the melting point of bulk EMI-TFSA (257 K), as shown in the DSC data. By contrast, the ionic conductivity of EZ50, EZ75, and EZ100 showed no sharp decrease corresponding to the phase transition on freezing between 228 and 341 K. The EMI-TFSA inside the ZIF-8 micropores is thus thought to remain liquid, even in the low temperature region. Therefore, the ionic conductivity is maintained in the temperature range where bulk EMI-TFSA is frozen. EZ100 showed a higher ionic conductivity compared with bulk EMI-TFSA below 250 K. This result indicates that IL@MOF could be used as an electrolyte for electrochemical devices that operate in the low temperature region. In addition, the ionic conductivity of EMI-TFSA@ZIF-8 could be increased by several orders of magnitude, dependent on the concentration of EMI-TFSA. This result is anomalous, because in general, ionic conductivity increases linearly with the number of conducting ions present.

**Fig. 6:**
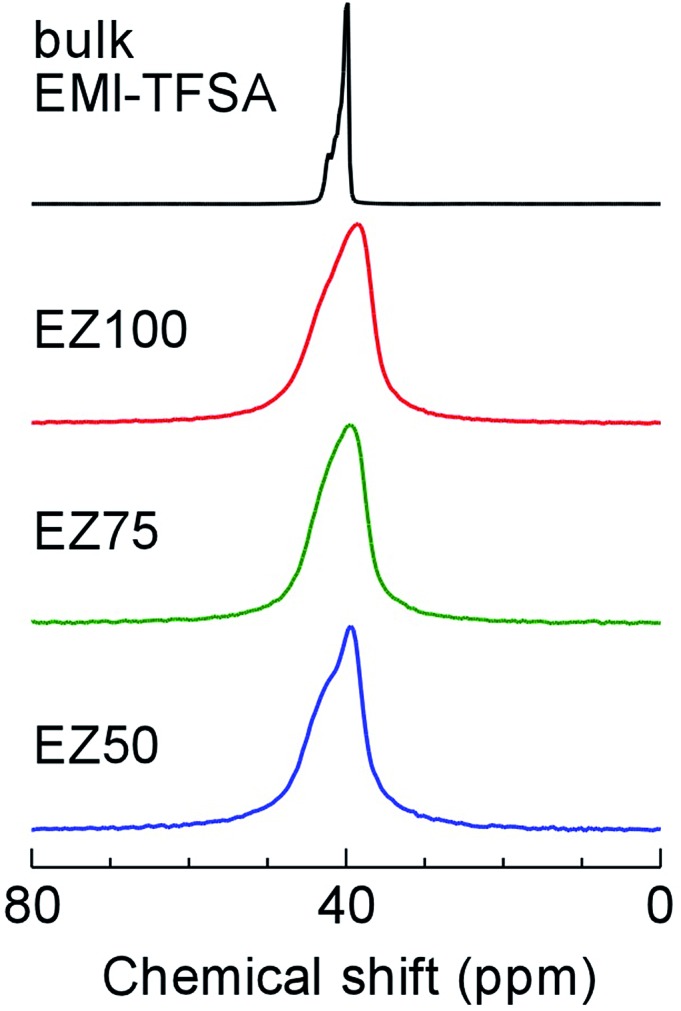
Solid-state ^19^F static NMR spectra of EZ50, EZ75, EZ100, and bulk EMI-TFSA at ambient temperature.

We carried out solid-state ^19^F static nuclear magnetic resonance (NMR) measurements to study the origin of the strong dependence of the conductivity on the concentration of EMI-TFSA. Fig. 6 shows ^19^F NMR spectra at ambient temperature, indicating the motion state of the TFSA^–^ anions. A sharp line was observed in the spectrum of bulk EMI-TFSA. This indicates a “motional narrowing” arising from the free rotation and diffusion of the TFSA^–^ anions in the bulk liquid state. In all the EMI-TFSA@ZIF-8 samples, almost the same spectrum was obtained, even though the ionic conductivity had increased by up to several orders of magnitude, dependent on the concentration of EMI-TFSA. Therefore, we hypothesized that the strong EMI-TFSA concentration dependence of the ionic conductivity in EMI-TFSA@ZIF-8 does not originate from the mobility of the guest ions, assuming that the mobility of the EMI^+^ cations and TFSA^–^ anions was in the same range as in bulk EMI-TFSA.^21^ One of the most reasonable explanations for this observation is that the conductive paths for the ions were strongly connected with an increasing concentration of EMI-TFSA ions. Such continuous paths for mobile ions would lead to the high ionic conductivity of IL@MOF.

## Conclusions

We have demonstrated the ionic conductivity of an IL inside the micropores of a MOF for the first time. EMI-TFSA inside ZIF-8 micropores showed no marked phase transition from DSC measurements. EMI-TFSA@ZIF-8 showed no marked decrease in conductivity between 228 and 341 K, and showed higher conductivity than bulk EMI-TFSA below 250 K because the nanosized IL in the micropores was prevented from undergoing the freezing transition. This result provides a route towards developing novel electrolytes for electrochemical devices such as secondary batteries and EDLCs that could operate in the low temperature region.
