# Supplementary material for: Low temperature ionic conductor: ionic liquid incorporated within a metal–organic framework
Source: Chem Sci. 2015 May 5;6(7):4306–10. doi: 10.1039/c5sc01398d (PMC5707510; doi:10.1039/c5sc01398d)
Supplement: Supplementary file 1 [file SC-006-C5SC01398D-s001.pdf]

Electronic Supplementary Material (ESI) for Chemical Science.
This journal is © The Royal Society of Chemistry 2015

Supplementary Information

Low Temperature Ionic Conductor: Ionic Liquid Incorporated within a Metal–Organic Framework

Kazuyuki Fujie*[ab], Kazuya Otsubo[b], Ryuichi Ikeda[bc], Teppei Yamada†[b] and Hiroshi Kitagawa*[bdef]

[a] R&D Center Kagoshima, Kyocera Corporation, 1-4 Kokubuyamashita-cho, Kirishima-shi, Kagoshima 899-4312, Japan

[b] Division of Chemistry, Graduate School of Science, Kyoto University, Kitashirakawa Oiwake-cho, Sakyo-ku, Kyoto 606-8502, Japan

[c] Department of Chemistry, University of Tsukuba, Tsukuba 305, Japan

[d] Core Research for Evolutional Science and Technology (CREST), Japan Science and Technology Agency (JST), 5 Sanban-cho, Chiyoda-ku, Tokyo 102-0075, Japan

[e] Institute for Integrated Cell-Material Sciences (iCeMS), Kyoto University, Yoshida, Sakyo-ku, Kyoto 606-8501, Japan

[f] INAMORI Frontier Research Center, Kyushu University, 744 Motooka, Nishi-ku, Fukuoka 819-3095, Japan

†Present addresses: Department of Chemistry and Biochemistry, Graduate School of Engineering, Kyushu University, 744 Moto-oka, Nishi-ku, Fukuoka 819-0395, Japan; International Research Center for Molecular Systems (IRCMS), Kyushu University, 744 Moto-oka, Nishi-ku, Fukuoka 819-0395, Japan

1. Materials and methods

Synthesis of ZIF-8.

A methanol solution (75 mL) of $Zn(NO_3)_2$•$6H_2O$ (1.10 g, 3.70 mmol) was added to a methanol solution (75 mL) of H(MeIM) (1.22 g, 14.8 mmol) and triethylamine (3.00 g, 29.7 mmol) at ambient temperature. The mixture was kept at room temperature for a period of 1 h with stirring. The resulting white-colored precipitate was collected using centrifugation, and washed five times using methanol. The ZIF-8 nanoparticles obtained were dried at 423 K overnight under vacuum.

Synthesis of EMI-TFSA@ZIF-8.

The EMI-TFSA used was purchased from the Tokyo Chemical Industry Co. Ltd and used without further purification. The EMI-TFSA was mixed with dried ZIF-8 using a mortar and pestle with molar ratios of 0.14:1, 0.28:1, 0.42:1, 0.57:1, and 0.71:1, at which EMI-TFSA theoretically occupied the volume of the micropores at loadings of 25%, 50%, 75%, 100%, and 125%, respectively (denoted as EZ25, EZ50, EZ75, EZ100, and EZ125, respectively). These occupancies were estimated by using the pore volume of ZIF-8 (0.636 cm^3 g^{-1})[S1] and the density of EMI-TFSA (1.520 g cm^{-3})[S2]. The mixtures were heated and stored at 423 K overnight to enhance the diffusion of EMI-TFSA into the micropores of the ZIF-8. All preparation processes were carried out in a glove box filled with dried argon gas to prevent water adsorption onto the ZIF-8 and water absorption into the EMI-TFSA.

Structural Characterizations

XRPD measurements were performed using a Bruker D8 ADVANCE diffractometer (λ = 1.54059 Å, CuKα radiation) in air. Maximum entropy method (MEM)/Rietveld analysis was conducted in the following method to obtain electron density distribution inside micropores of ZIF-8. Note that bulk sample of ZIF-8 (denoted as ZIF-8_{bulk}; commercially available Basolite® Z1200) was used as the host material only for the MEM/Rietveld analysis to obtain the XRPD patterns with sufficiently high signal/noise ratio. Rietveld refinement was applied for ZIF-8_{bulk}, $EZ25_{bulk}$ and $EZ100_{bulk}$ with RIETAN-FP[S3], using the reported crystal structure data as the initial model of the ZIF-8 framework.[S1] Simulated annealing (SA) method was applied for $EZ25_{bulk}$ and $EZ100_{bulk}$ using EXPO2013 before Rietveld analysis. Electron density distribution was obtained by MEM with Dysnomia[S4].

Physical measurements.

Nitrogen gas adsorption and desorption measurements were carried out at 77 K using an automatic gas adsorption apparatus, BELSORP-max (BEL Japan). In the gas sorption measurements, the samples were sealed in glass tube in a glove box to prevent water adsorption or absorption. DSC measurements were performed using a DSC3100SA (Netzsch Japan). In the DSC measurements, the samples were hermetically sealed in aluminium pans in a glove box. The samples were first heated to 473 K, then allowed to cool to 143 K, and

then reheated to 473 K. The scan rate was fixed at 5 K min^{-1}. The ac impedance measurements were carried out using a quasi-four-probe method using a Solartron 1260 Impedance/Gain-Phase Analyzer and a 1296 Dielectric Interface System in the frequency range 1 Hz–1 MHz. For the impedance measurements, EMI-TFSA@ZIF-8 powder was pressed into a pellet (approx. 0.5 mm thick × 3.0 mm in diameter) with carbon sheet electrodes attached to both faces using hand press instrument. The pressed pellet was sandwiched between stainless steel electrodes in a homemade cryostat filled with dried argon gas. Bulk EMI-TFSA samples were placed in a Kapton washer (approx. 0.1 mm thick × 3.1 mm inner diameter) and sandwiched between stainless steel electrodes. The samples were first cooled to below 210 K, and then measured in steps during heating. Solid-state ^{19}F static NMR measurements were performed at ambient temperature at 376.5 MHz using a Bruker AVANCE II^{+} 400 spectrometer. The samples were hermetically sealed in glass tubes for the NMR measurements. The NMR spectra were obtained by applying a Fourier transform to the resulting free induction decay (FID) signal after a single $\pi/2$ pulse sequence. IR spectra (Fig. S13) were obtained using the attenuated total reflectance (ATR) method on an ALPHA FT-IR spectrometer with a platinum ATR module (Bruker) in a glove box. The spectra were obtained from 400 to 4000 cm^{-1} with a resolution of 2 cm^{-1}.

2. Results and discussion

Maximum-Entropy Method (MEM)/Rietveld Analysis for EMI-TSFA loaded ZIF-8

As shown in Fig. 1 in the main text, EMI-TFSA loading provided clear changes in relative intensity in X-ray powder diffraction (XRPD) patterns. To obtain structural information of EMI-TFSA loaded ZIF-8 samples, structural analyses based on the Rietveld refinement were performed. Because very weak diffractions with insufficient signal/noise ratio were observed in present nanocrystal samples, bulk samples of ZIF-8 (ZIF-8_{bulk}) and EMI-TFSA loaded ZIF-8 with varying loading amount ($EZ25_{bulk}$ and $EZ100_{bulk}$) were used for structural analysis. Fig. S4 shows the XRPD patterns of activated ZIF-8_{bulk}, $EZ25_{bulk}$, and $EZ100_{bulk}$[S5]. As shown in Fig. S4, spontaneous change in diffraction intensity ratio was observed by EMI-TFSA loading, where intensities of 011 and 013 Bragg peaks were changed remarkably. This tendency of profile change in bulk state is almost same as seen in those of nanocrystals (Fig. 1). Similar change in intensity ratio have been reported alkylammonium salt included ZIF-8[S6]. Therefore, this result suggests that ionic liquid EMI-TFSA was successfully included inside the pore of ZIF-8. Powder indexing and Pawley refinement using EXPO2013 program[S7] on EMI-TFSA loaded samples led to the similar lattice constants with same space group (a ~ 17 Å, cubic I–43m), which indicated that the original cage structure of ZIF-8 is retained even after EMI-TFSA loading. To evidence the EMI-TSFA inclusion in ZIF-8, we performed maximum-entropy method (MEM)/Rietveld analysis on present materials. It is well known that MEM analysis is useful technique for visualising electron density of guest molecule included in porous materials[S6,S8,S9].

Firstly, activated ZIF-8 sample in bulk state was analysed using MEM/Rietveld method with programs RIETAN-FP, Dysnomia, and VESTA[S10]. The ZIF-8 with 'empty cage (i.e. without guest)' structure based on the single-crystal analysis[S1] was used as initial structural model. Fig. S5a shows profile fitting result based on Rietveld refinement for activated ZIF-8. By using empty cage model of ZIF-8, good fitting result was obtained with favourable reliability (R) factors related to weighted profile, Bragg integrated intensity, and structural factor (R_{wp}, R_B, and R_F). The MEM analysis was carried out using structure factors obtained from Rietveld analysis (Fig. S5a). Fig. S5b shows MEM equi-charge-density surface (isosurface level: 0.15 eÅ^{-3}) of activated ZIF-8 in the selected range ($0.24 \leq x \leq 0.76$, $0 \leq y \leq 1$, and $0 \leq z \leq 1$, where we can see the charge density inside the cage.). The result clearly shows that the activated ZIF-8 contains no guest molecules.

Next EMI-TFSA loaded sample ($EZ25_{bulk}$) was examined. Preliminary Rietveld fitting result of $EZ25_{bulk}$ based on the 'empty cage' model same as activated ZIF-8 is shown in Fig. S5c. As is clearly seen, empty cage model of ZIF-8 could not reproduce the experimental diffraction data. Fig. S5d shows preliminary MEM equi-charge-density surface (isosurface level: 0.15 eÅ^{-3}) of $EZ25_{bulk}$ in the same range as Fig. S5b. In contrast to the case of activated ZIF-8, some electron density peaks which could not be assigned to the host ZIF-8 framework were also found in the cage despite no assumption of guest molecules. This result suggested that ionic liquid EMI-TFSA molecules are included in the cage of ZIF-8 framework. However, we could not model the EMI and TFSA molecules inside the cage using MEM equi-charge density surface because of low symmetry of guest molecules within the highly symmetric host ZIF-8 framework. Therefore, ab initio structure

solution was carried out by simulated annealing method installed on EXPO2013 program. Considering the highly symmetric space group of ionic liquid EMI-TFSA loaded samples ($EZ25_{bulk}$ and $EZ100_{bulk}$), the ZIF-8 structure with one pair of EMI and TFSA molecules within the cage was used as initial structure model followed by varying occupancy factor of EMI-TFSA part. For EMI and TFSA parts, several constraints were applied to bond length, bond angle, rotation, and torsion angle, whereas host ZIF-8 framework was fixed in simulated annealing calculation. A total of 30 cycles calculation which consists of each ~5×10^6 Monte Carlo moves of EMI and TFSA molecules gave initial structural models with good *R* factors favourable for final Rietveld refinement.

Figs. S6(a) and (b) shows Rietveld fitting results of $EZ25_{bulk}$ and $EZ100_{bulk}$ based on the EMI-TFSA included models. By using "guest included" models, good fitting results were obtained with favourable *R* factors (R_{wp}, R_B, and R_F). These results clearly indicated that ionic liquid EMI-TFSA units are surely included inside the cage of ZIF-8. The crystal structures of EMI-TFSA included ZIF-8 are shown in Fig. S7. Number of pairs of EMI and TFSA units from Rietveld refinement were calculated to be 0.46 pairs/cage for $EZ25_{bulk}$ and 3.0 pairs/cage for $EZ100_{bulk}$, respectively, which are consistent with the empirical values based on the molar ratio in preparation (0.84 pairs/cage for $EZ25_{bulk}$, 3.4 pairs/cage for $EZ100_{bulk}$). Then the MEM analyses on both samples were carried out using structure factors obtained from Rietveld refinements. Figs. S6(c) and (d) shows MEM equi-charge-density surfaces of $EZ25_{bulk}$ and $EZ100_{bulk}$ in the same range as that of Fig. S8b. Obvious electron density peaks assignable to the EMI-TFSA were found in the cage of ZIF-8 framework, which are widely spread inside the cage due to the disordered nature. 2D MEM charge-density contour plots of present materials are also shown in Fig. S8. As is clearly seen from Fig. S8, electron density within the cage spontaneously increase with increasing loading amount of EMI-TFSA. From the results of MEM/Rietveld analysis, we can conclude that ionic liquid EMI-TFSA is successfully included inside the pore of ZIF-8 framework.

Final Rietveld refinement result of ZIF-8$_{\mathbf{bulk}}$ ($C_8H_{10}N_4Zn$): Cubic, Space Group *I*–43m, *a* = 17.013(3) Å, *V* = 4924(1) Å^3, *Z* = 12, R_{wp} = 9.00%, R_B = 3.56%, R_F = 2.03%, *S* = 2.46, *R* factor of MEM analysis R_F(MEM) = 3.13%.

Final Rietveld refinement result of EZ25$_{\mathbf{bulk}}$ ($C_{8.61}H_{10.84}F_{0.456}N_{4.23}O_{0.456}S_{0.152}Zn$): Cubic, Space Group *I*–43m, *a* = 16.980(3) Å, *V* = 4895(1) Å^3, *Z* = 12, R_{wp} = 7.16%, R_B = 1.65%, R_F = 1.35%, *S* = 3.25, R_F(MEM) = 1.24%, CCDC reference number 1058658.

Final Rietveld refinement result of EZ100$_{\mathbf{bulk}}$ ($C_{12}H_{17.75}F_3N_{5.5}O_2SZn$): Cubic, Space Group *I*–43m, *a* = 17.038(2) Å, *V* = 4946(1) Å^3, *Z* = 12, R_{wp} = 6.89%, R_B = 4.07%, R_F = 4.12%, *S* = 1.50, R_F(MEM) = 1.81%, CCDC reference number 1058659.

3. Figures

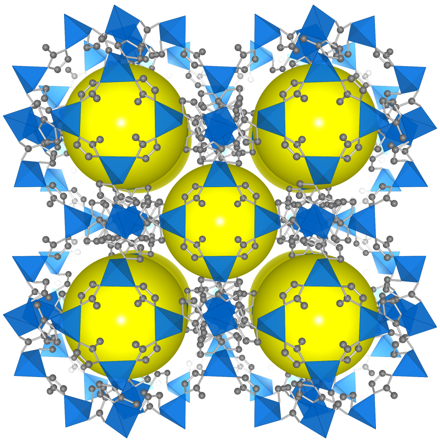

Fig. S1 Crystal structure of ZIF-8,[S1] where blue tetrahedra, gray balls, and yellow spheres indicate ZnN_4, C, and micropores, respectively. Hydrogen atoms are omitted for clarity.

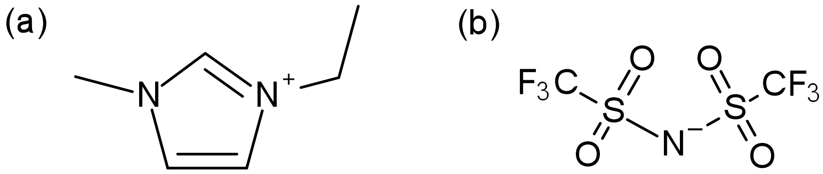

Fig. S2 Structure of EMI-TFSA (1-ethyl-3-methylimidazolium bis(trifluoromethylsulfonyl)amide). EMI^+ cation (a) and $TFSA^-$ anion (b).

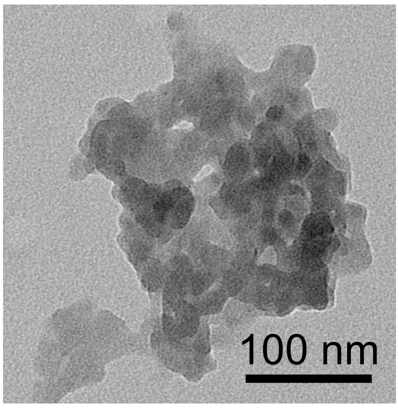

Fig. S3 TEM image of the synthesized ZIF-8 nanoparticles.

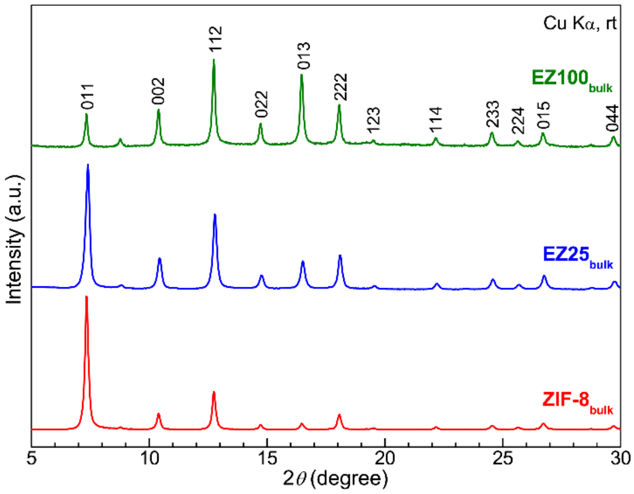

Fig. S4 XRPD patterns of ZIF-8_{bulk} (red), $EZ25_{bulk}$ (blue), and $EZ100_{bulk}$ (green).

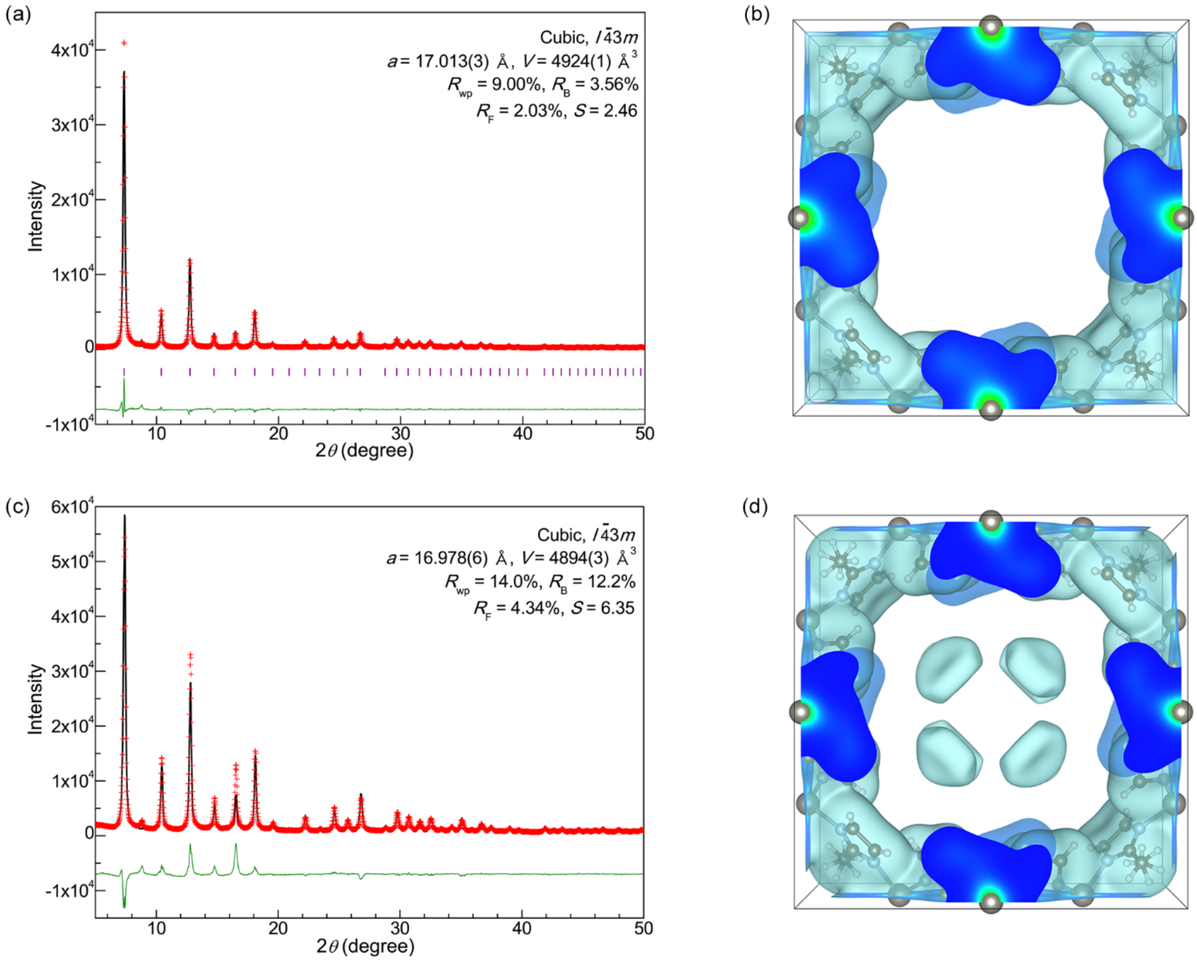

Fig. S5 Rietveld fitting result (a) and MEM equi-charge density surface (b, isosurface level: 0.15 e$Å^{-3}$) of ZIF-8_{bulk}. Preliminary Rietveld fitting result (c) and MEM equi-charge density surface (d, isosurface level: 0.15 e$Å^{-3}$) of $EZ25_{bulk}$ based on the "empty cage" model. In panels (a) and (c), red crosses, thick black line, thin green line, and violet ticks denote experimental, calculated, difference profiles, and positions of Bragg peaks, respectively.

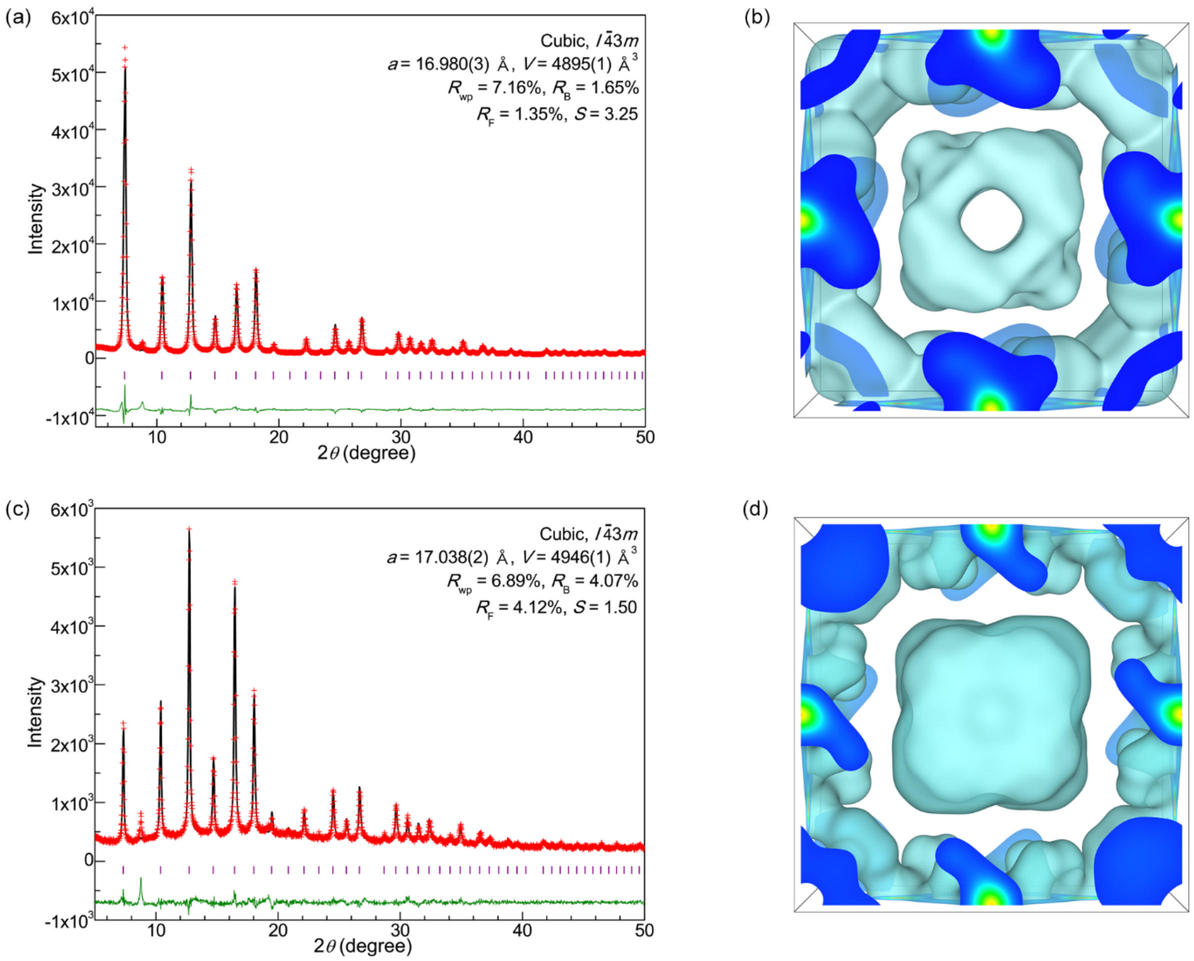

Fig. S6 Final Rietveld fitting result (a) and MEM equi-charge density surface (b, isosurface level: 0.15 eÅ^{-3}) of EZ25$_{bulk}$. Final Rietveld fitting result (c) and MEM equi-charge density surface (d, isosurface level: 0.50 eÅ^{-3}) of EZ100$_{bulk}$. In panels (a) and (c), red crosses, thick black line, thin green line, and violet ticks denote experimental, calculated, difference profiles, and positions of Bragg peaks, respectively.

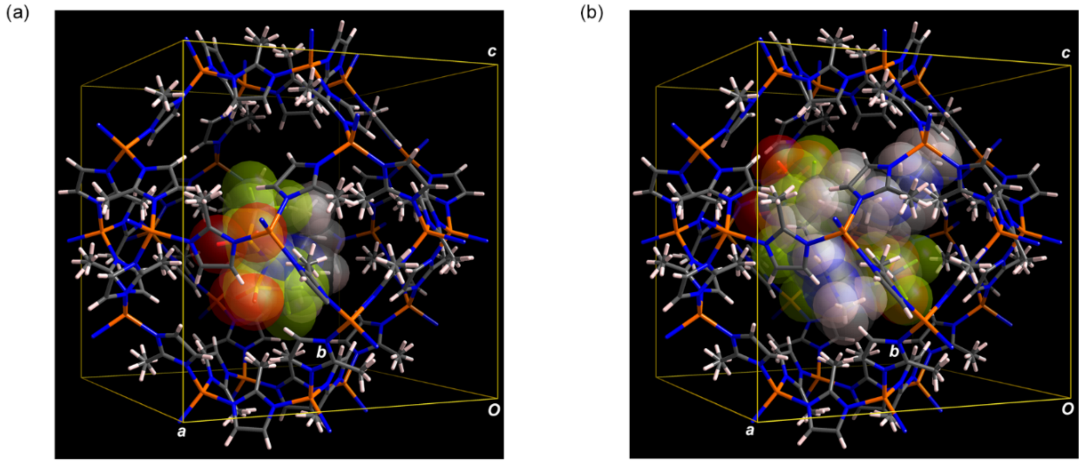

Fig. S7 Crystal structures of $EZ25_{bulk}$ (a) and $EZ100_{bulk}$ (b) obtained from Rietveld refinement. Included EMI-TFSA units are drawn by stick model with superimposed CPK model. Zinc, carbon, nitrogen, oxygen, fluorine, sulphur and hydrogen atoms are shown in orange, grey, blue, red, green, yellow, and pink, respectively.

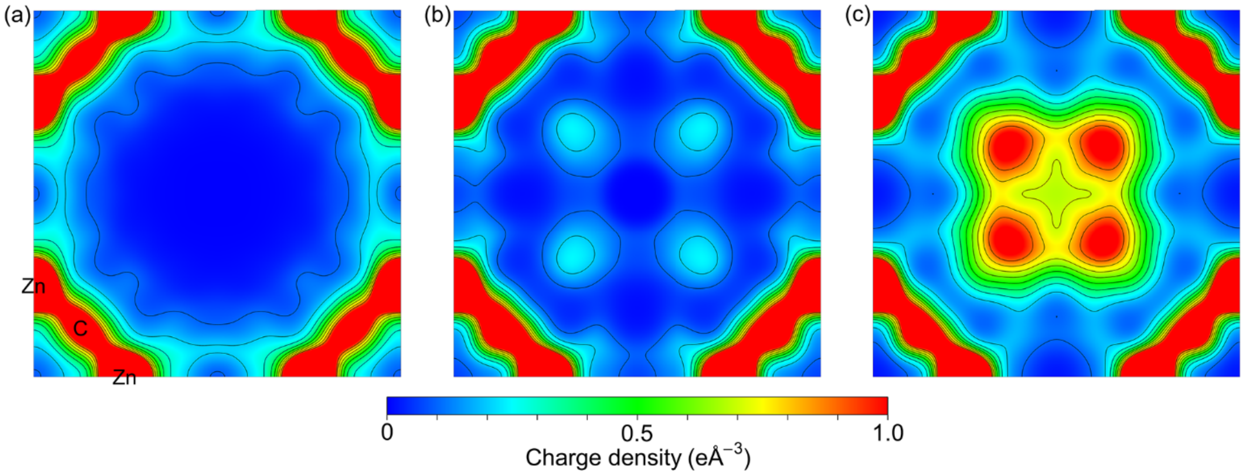

Fig. S8 2D contour maps of MEM charge density of ZIF-8_{bulk} (a), $EZ25_{bulk}$ (b), and $EZ100_{bulk}$ (c) sliced in (200) plane. All contour maps are drawn from 0 to 1.0 eA^{-3} at intervals of 0.1 eA^{-3}.

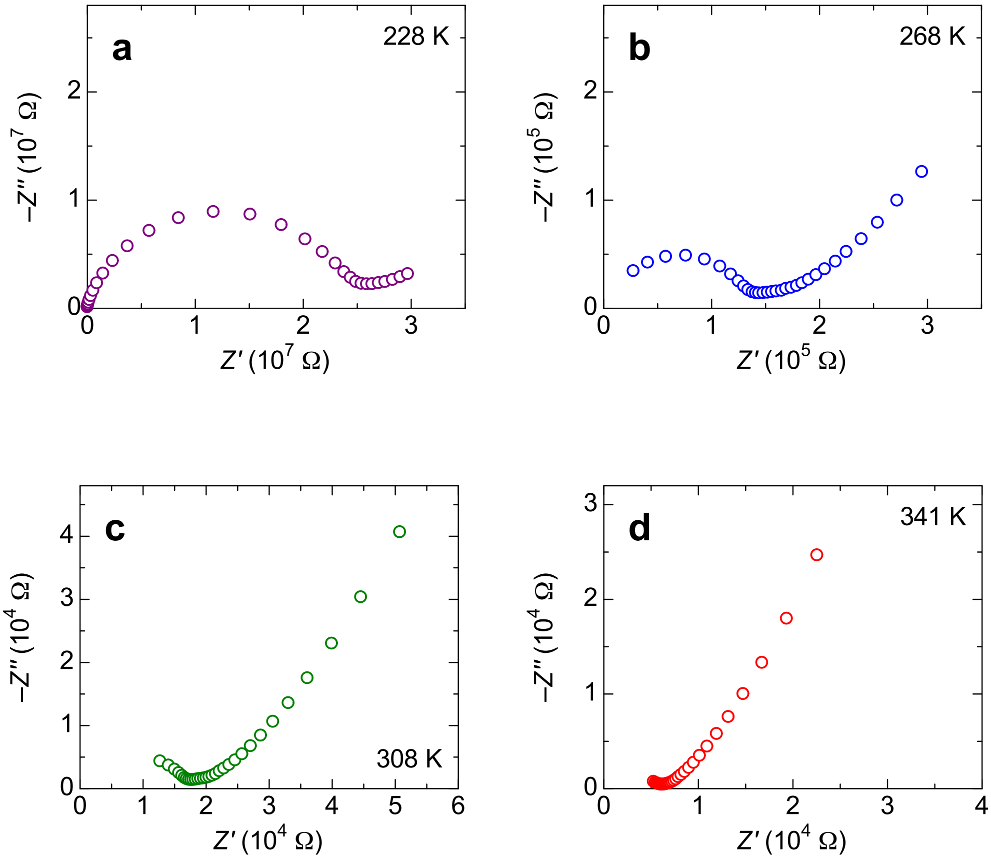

Fig. S9 Nyquist plots of EZ100. 228 K (a), 268 K (b), 308 K (c), and 341 K (d).

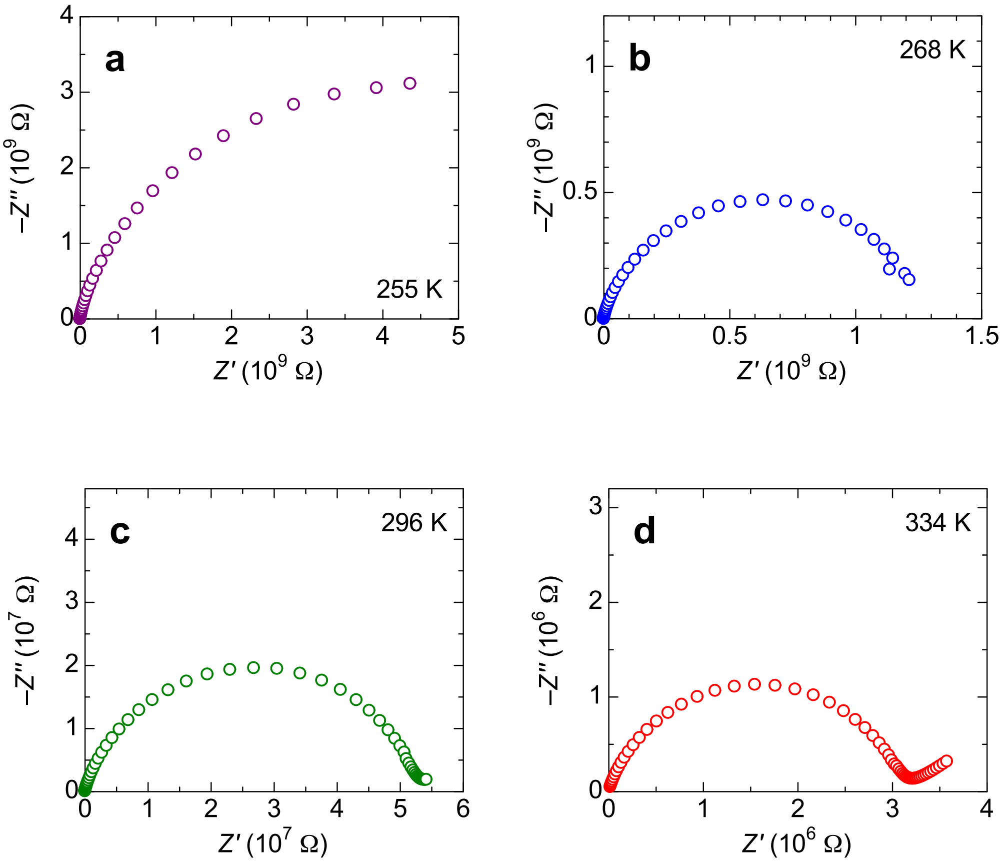

Fig. S10 Nyquist plots of EZ75. 255 K (a), 268 K (b), 296 K (c), and 334 K (d).

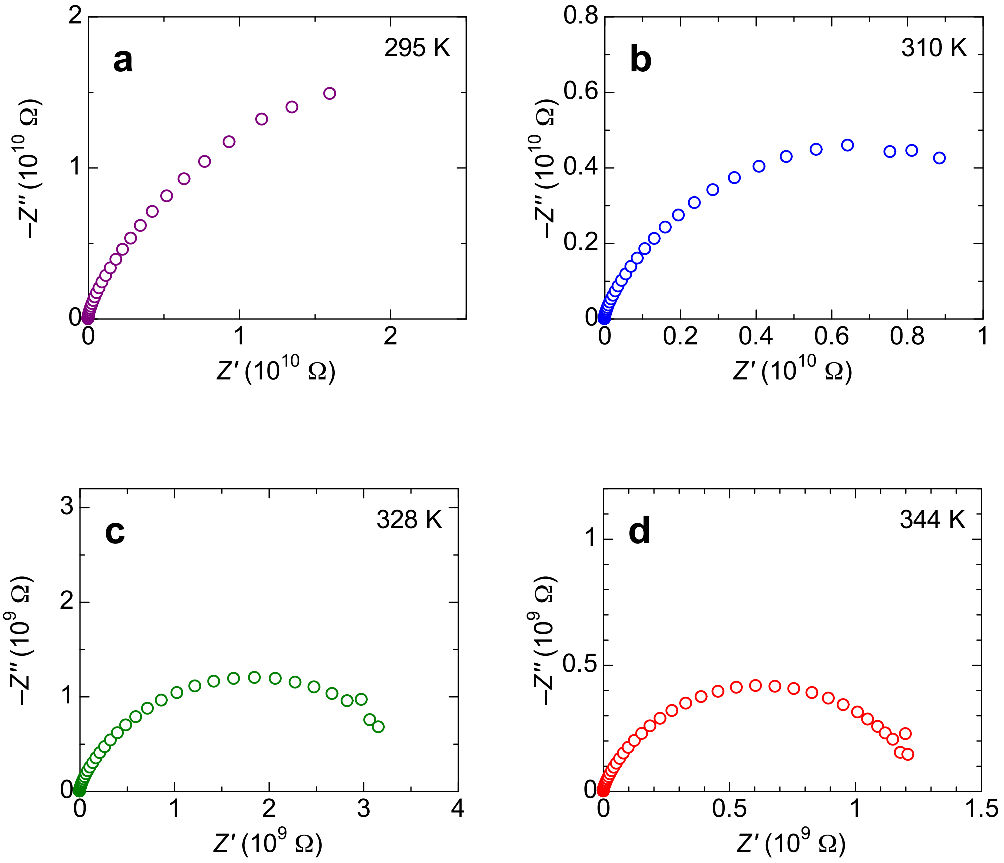

Fig. S11 Nyquist plots of EZ50. 295 K (a), 310 K (b), 328 K (c), and 344 K (d).

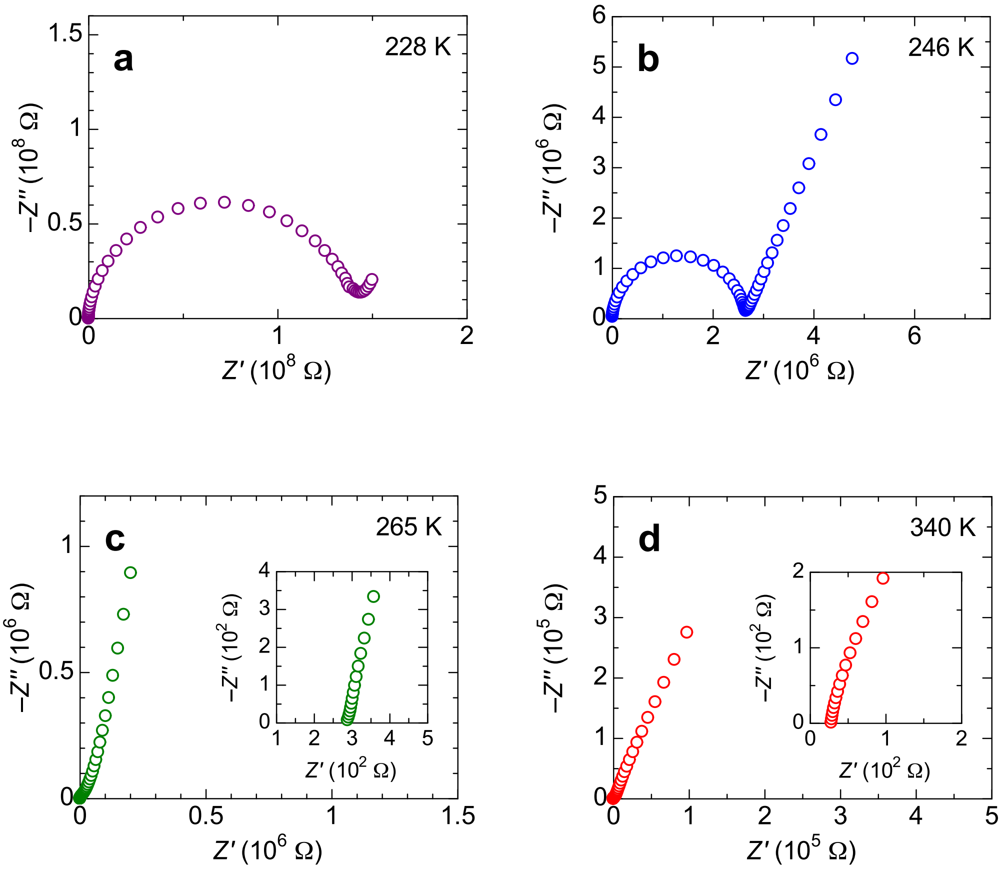

Fig. S12 Nyquist plots of bulk EMI-TFSA. 228 K (a), 246 K (b), 265 K (c), and 340 K (d).

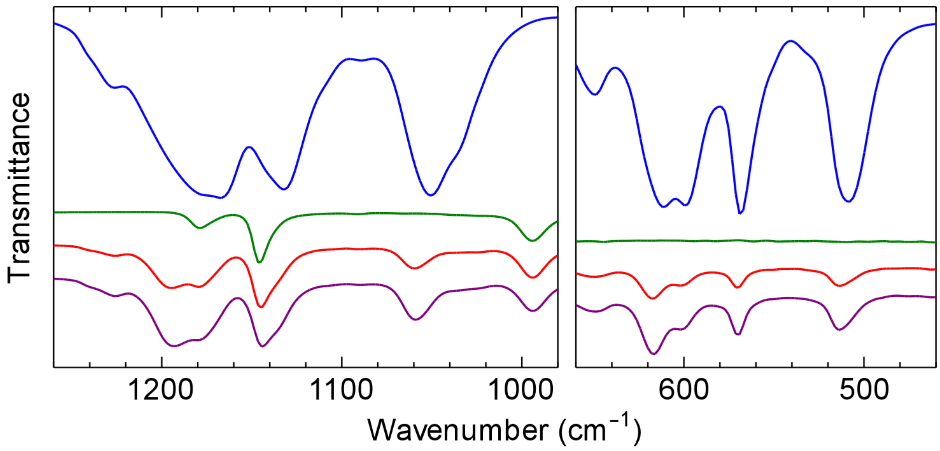

Fig. S13 IR spectra of bulk EMI-TFSA (blue), ZIF-8 (green), EZ25 (red), and EZ50 (purple). Peaks at 1059, 618, and 514 cm^{-1} in EZ25 and EZ50 can be assigned to EMI-TFSA. These peaks showed blue shifts from the peaks of the bulk EMI-TFSA. The blue shifts can be explained by the attractive interaction of the ions with the inner surface of the micropores as the result of introduction into the micropores.

Table S1 Results of elemental analysis for ZIF-8, EZ50, and EZ100. Calculated values are based on the molar ratio in preparation.

Samples		C	H	N
ZIF-8	Calcd.	42.22	4.44	24.63
	Found	41.34	4.31	24.42
EZ50	Calcd.	36.44	3.91	20.08
	Found	35.56	3.81	20.07
EZ100	Calcd.	33.51	3.65	17.78
	Found	32.91	3.56	17.73

4. References

S1 K. S. Park, Z. Ni, A. P. Côté, J. Y. Choi, R. Huang, F. J. Uribe-Romo, H. K. Chae, M. O'Keeffe and O. M. Yaghi, *Proc. Natl. Acad. Sci. U.S.A.*, 2006, **103**, 10186.

S2 P. Bonhôte, A.-P. Dias, N. Papageorgiou, K. Kalyanasundaram and M. Grätzel, *Inorg. Chem.* 1996, **35**, 1168.

S3 F. Izumi and K. Momma *Solid State Phenom.*, 2007, **130**, 150.

S4 K. Momma, T. Ikeda, A.A. Belik and F. Izumi, *Powder Diffr.*, 2013, **28**, 184.

S5 K. Fujie, T. Yamada, R. Ikeda and H. Kitagawa, *Angew. Chem. Int. Ed.*, 2014, **53**, 11302.

S6 M. Sadakiyo, H. Kasai, K. Kato, M. Takata, and M. Yamauchi, *J. Am. Chem. Soc.*, 2014, **136**, 1702.

S7 A. Altomare, C. Cuocci, C. Giacovazzo, A. Moliterni, R. Rizzi, N. Corriero and A. Falcicchio, *J. Appl. Cryst.*, 2013, **46**, 1231.

S8 R. Matsuda, R. Kitaura, S. Kitagawa, Y. Kubota, R. V. Belosludov, T. C. Kobayashi, H. Sakamoto, T. Chiba, M. Takata, Y. Kawazoe and Y. Mita, *Nature*, 2005, **436**, 238.

S9 D. Fujita, K. Suzuki, S. Sato, M. Yagi-Utsumi, Y. Yamaguchi, N. Mizuno, T. Kumasaka, M. Takata, M. Noda, S. Uchiyama, K. Kato and M. Fujita, *Nat. Commun.*, 2010, **3**, 1093.

S10 K. Momma and F. Izumi, *J. Appl. Crystallogr.* 2008, **41**, 653.
